# Nanodelivery strategies for the treatment of multidrug‐resistant bacterial infections

**DOI:** 10.1002/jin2.48

**Published:** 2018-09-04

**Authors:** Lai Jiang, Jia Lin, Clifford C. Taggart, José A. Bengoechea, Christopher J. Scott

**Affiliations:** ^1^ Centre for Experimental Medicine, School of Medicine, Dentistry and Biomedical Sciences Queen's University Belfast Belfast UK; ^2^ Centre for Cancer Research and Cell Biology, School of Medicine, Dentistry and Biomedical Sciences Queen's University Belfast Belfast UK; ^3^ School of Pharmacy Queen's University Belfast Belfast UK

**Keywords:** Antibiotics, ESKAPE, infection, intracellular, nanoparticles

## Abstract

One of the most important health concerns in society is the development of nosocomial infections caused by multidrug‐resistant pathogens. The purpose of this review is to discuss the issues in current antibiotic therapies and the ongoing progress of developing new strategies for the treatment of ESKAPE pathogen infections, which is acronymized for *Enterococcus faecium*, Staphylococcus aureus, Klebsiella pneumoniae, *Acinetobacter baumannii*, *Pseudomonas aeruginosa*, and *Enterobacter* species. We not only examine the current issues caused by multidrug resistance but we also examine the barrier effects such as biofilm and intracellular localization exploited by these pathogens to avoid antibiotic exposure. Recent innovations in nanomedicine approaches and antibody antibiotic conjugates are reviewed as potential novel approaches for the treatment of bacterial infection, which ultimately may expand the useful life span of current antibiotics.

## Introduction

Since the first discovery and development of antibiotics, successful treatment of bacterial infectious diseases was achieved in a rapid timescale. However, along with the broad‐spread use of antibiotics, the persistence of bacterial infections caused by multidrug‐resistant (MDR) bacteria now poses a significant public health challenges worldwide.

In 2008, the Infectious Diseases Society of America proposed the acronym ESKAPE to standardize a group of deadly bacterial pathogens with rapidly growing MDR properties, including *Enterococcus faecium*, Staphylococcus aureus, Klebsiella pneumoniae, *Acinetobacter baumannii*, *Pseudomonas aeruginosa*, and *Enterobacter* species (Rice, 2008). These ESKAPE pathogens were able to “escape” from the biocidal action of currently marketed antibacterial drugs, causing increasingly serious life‐threatening nosocomial infections, also known as hospital‐acquired infections (HAIs) (Boucher et al., 2009; Slavcovici et al., 2015).

The English National Point Prevalence Survey (Health Protection Agency 2012) identified that 6.4% of inpatients in 2011 had HAIs in the UK. The Centers for Disease Control and Prevention estimated that over 2 million infections and about 23,000 deaths per year worldwide were caused by antibiotic‐resistant ESKAPE pathogens (Najafi et al., 2016). In recent years, the HAIs problem has caused great concern all over the world, but with the collaboration of the Infectious Diseases Society of America, the European Centre for Disease Prevention and Control (ECDC), and the World Health Organization (WHO), pharmaceutical industries were encouraged to reinvestigate their research with a focus on novel antimicrobial combinations to treat infections caused by ESKAPE pathogens (Boucher et al., 2009; European Commission, 2011; WHO, 2015).

However, despite these ongoing efforts and some notable successes, our therapeutic options for ESKAPE pathogens, such as vancomycin‐resistant *E*. *faecium*, fluoroquinolone‐resistant *P*. *aeruginosa*, and carbapenem‐resistant *Klebsiella* species, are extremely limited. Therefore, further understanding of resistance mechanisms in these pathogens may lead to innovative strategies for the development of new antimicrobial options.

## The Issue of Current Therapy

### Antibiotic resistance in ESKAPE pathogens

#### Enterococcus faecium

*Enterococcus* species are Gram‐positive facultative anaerobes, in which *E*. *faecium* is the most clinically relevant implicated in HAIs (Ciftci et al., 2009; Silva et al., 2011). These *E*. *faecium* infections account for approximately 40% of all enterococcal infections currently (Gao et al., 2018). And recently, increasing resistance of *E*. *faecium* to beta‐lactam antibiotics has been reported in health‐care facilities (Chen et al., 2015). Among them, the vancomycin‐resistant *E*. *faecium* isolate has emerged in North America during the late 1980s, with an alarming increase in resistance of 61% by 2002 (Cetinkaya et al., 2000). In the UK and Ireland, it was reported by the British Society and Antimicrobial Chemotherapy that the incidence of vancomycin‐resistant *E*. *faecium* had risen from approximately 20% to over 30% in the period 2001 to 2006 (Brown et al., 2008). Nine different types of vancomycin resistance genes in *Enterococci* (Van‐A to E, G, L, M, and N) have been reported, in which Van‐A was the most prevalent worldwide, showing the highest resistance to all glycopeptides (Cattoir et al., 2013; Protonotariou et al., 2010).

#### Staphylococcus aureus

*Staphylococcus aureus* is a Gram‐positive coccal bacterium, which is present in around 20% of healthy individuals (Kluytmans et al., 1997; Zeeuwen et al., 2018). Because of the excessive use of beta‐lactam antibiotics, *Staphylococcus* species possess a method of gene transfer to produce beta‐lactamase positive isolates, resulting in 65–85% staphylococcal clinical isolates being resistant to penicillin G (Santajit et al., 2016). Methicillin‐resistant S. aureus (MRSA) is defined as a strain of S. aureus that had developed resistance to beta‐lactam antibiotics, including all penicillin, cephalosporins, and carbapenems. Currently, MRSA accounts for about 25% of S. aureus isolates, and in some regions, the prevalence is greater than 50% (ECDC, 2011). According to the Centers for Disease Control and Prevention, there are 80,000 cases and 11,000 deaths due to invasive MRSA strains each year (Najafi et al., 2016). In the clinic, glycopeptide antibiotics such as vancomycin were always used as the first choice treatment for MRSA infections. However, since the first reported resistant strains in Japan in the mid‐1990s, these MDR strains have now emerged all over the world (Chambers et al., 2009).

#### Klebsiella pneumoniae

As a member of the Enterobacteriaceae family, K. pneumoniae, a Gram‐negative bacterium, frequently causes lower respiratory tract infection and catheter‐associated urinary tract infection (Navon‐Venezia et al., 2017). Because of the presence of extended‐spectrum beta‐lactamases (ESBLs), K. pneumoniae is not only simply resistant to penicillin and ampicillin but also increasingly multidrug resistance to cephalosporin and ceftazidime (Petrosillo et al., 2013). Historically, carbapenems were typically used to treat only the most difficult MDR infections caused by Gram‐negative bacteria (Bush, 2010). However, in recent years, carbapenem‐resistant *K*. *pneumonia*, harbouring the carbapenemase gene on resident plasmids, were reported to have become a significant obstacle to treatment by clinicians in many areas of the world and were associated with high rates of mortality (Bratu et al., 2005; Naas et al., 2005; Nordmann et al., 2009; Queenan et al., 2007; Wei et al., 2007). There are currently three types of K. pneumoniae producing carbapenemases (KPCs): KPC‐1, KPC‐2, and KPC‐3 (Yigit et al., 2001; Monteiro et al., 2009; Humphries et al., 2015). Currently, carbapenem‐resistant *K*. *pneumonia* with multidrug resistance only can be reduced and not completely eradicated; therefore, effective treatments are urgently needed to tackle this pathogen.

#### Acinetobacter baumannii

*Acinetobacter baumannii* is a non‐fermentative Gram‐negative opportunistic pathogen, with the ability to cause HAIs, particularly respiratory tract, urinary tract, and wound infections (Abbo et al., 2005; Al Mobarak et al., 2014; Fournier, 2006), the latter becoming increasingly prevalent in war areas, such as Iraq (Howard et al., 2012). In the period of January 2002 to August 2004, *A*. *baumannii* bloodstream infections were detected in 85 soldiers in Iraq and Afghanistan; among them, 35% of the strains were susceptible to only one type of antibiotic, and worryingly, 4% were resistant to all antibiotics (Montefour et al., 2008). This has created pressure on treatment options especially in those novel isolates with carbapenem resistance. In addition, imipenem metallo‐beta‐lactamases and oxacillinase serine beta‐lactamases, both of which are carbapenemases, have been discovered in *A*. *baumannii* isolates (Queenan et al., 2007; Vila et al., 2007). These strains show resistance not only to imipenem and colistin but also to traditional antimicrobial compounds, such as aminoglycosides, fluoroquinolones, and third generation of cephalosporins (Boucher et al., 2009; Fournier et al., 2006).

#### Pseudomonas aeruginosa

*Pseudomonas aeruginosa* is a Gram‐negative rod‐shaped facultative anaerobe, an opportunistic pathogen with a mortality rate of 40–60% (Najafi et al., 2016). P. aeruginosa is found in patients suffering the genetic condition cystic fibrosis (CF) (Mir et al., 2011). In CF patients, it has been found that P. aeruginosa survive in biofilms, thus creating an antibiotic‐resistant shield to treatment in the thick mucus of the CF lung. Recent reports documented that MDR P. aeruginosa was not only resistant to carbapenems, aminoglycosides, and quinolones, but also the polymyxins (Hirsch et al., 2011). A common MDR feature of P. aeruginosa is the combination of chromosomal AmpC production and upregulation of an efflux pump, which induce high‐level carbapenem resistance (Livermore, 2002). Additionally, P. aeruginosa can also produce ESBLs and other resistant enzymes such as KPC, imipenem metallo‐beta‐lactamases, and Verona integrin‐encoded metallo‐beta‐lactamases (BUSH et al., 1995; Livermore, 2002). The emergence of carbapenem resistance and MDR isolates represents a barrier to successful antibiotic therapies for the treatment of this microbe.

#### Enterobacter species

The genus *Enterobacter* is an important Gram‐negative nosocomial pathogen, causing an increasing number of serious HAIs, such as bloodstream infection and lower respiratory infection, resulting in MDR mediated by plasmid‐encoded ESBLs and carbapenemases (Verona integrin‐encoded metallo‐beta‐lactamases, KPC, and oxacillinase serine beta‐lactamases) (Castanheira et al., 2011). Other than colistin and tigecycline, these MDR *Enterobacter* species have developed resistance to almost all current available antimicrobials (Deshpande et al., 2006; Pfaller et al., 2006).

### Intracellular pathogens represent reservoirs of infection

In addition to the various mechanisms to develop resistance towards antimicrobial agents, recent studies have now shown another mechanism by which ESKAPE pathogens exhibit antibiotic resistance through the ability to survive intracellularly in host cells. Persistent bacterial infections caused by intracellular bacteria continue to impose significant challenges worldwide (Proctor et al., 2006). In order to maintain their infection cycle, certain pathogen species are able to localize inside host cells, creating a niche to reproduce and spread without damage to the host cells, leading to severe and latent infections (Imbuluzqueta et al., 2010; Silva et al., 2013; Hand et al., 2003).

Usually, phagocytes recognize and eliminate bacteria by phagocytosis. However, some bacteria can survive through various escape mechanisms. Recent research demonstrated that K. pneumoniae is engulfed by alveolar macrophages and resides inside the cell in a phagosome, called a Klebsiella‐contained vacuole (Cano et al., 2015). Whilst inside these vacuoles, K. pneumoniae promotes activation of Akt to arrest phagosome maturation and avoid fusion into lysosomes, which would otherwise result in destruction. Another ESKAPE pathogen, S. aureus, used to be considered as an extracellular bacterium; however, accumulating evidence suggests that S. aureus can invade and survive in either professional or nonprofessional phagocytes, including keratinocytes, endothelial cells, epithelial cells, fibroblast, and osteoblasts (Lacoma et al., 2017; Garzoni et al., 2009; Hanses et al., 2011; Reott et al., 2008). The adhesion of S. aureus to the host cell surface results in cytoskeletal rearrangement to allow S. aureus to move into cells. Once inside a cell, S. aureus can persist and even replicate within the acidic phagolysosome (Brouillette et al., 2003; Edwards et al., 2011). Moreover, other studies suggested another mechanism that some other bacteria, including S. aureus, can use to escape from the phagocytic vacuole to the cytosol (Fraunholz et al., 2012).

Furthermore, some other pathogens also exhibited similar mechanisms to maintain their life in host cells. For example, *Coxiella burnetii* infects mononuclear phagocytes, acquiring late endosomal‐early lysosomal markers, and resides inside the acidic vacuoles (Howe et al., 2010). *Mycobacterium tuberculosis* was reported recently that it can create its own vacuole to prevent phagosome‐lysosome fusion (Weiss et al., 2015) and also has the ability to escape into cytosol by permeabilizing the phagosome membranes (Jamwal et al., 2016).

As a consequence, professional phagocytes are not only unable to eradicate intracellular pathogens but provide a reservoir of latent infection that represents a significant barrier to successful treatment with antibiotics.

## Treatments for ESKAPE Pathogens

### Nano‐based drug delivery system

Macrophages are responsible for removing foreign pathogens or particles in the blood and tissue via phagocytotic pathways (Desjardins et al., 2003; Muppidi et al., 2011). However, phagocytes harbouring ESKAPE pathogens, such as *S*. *aureus* and K. pneumoniae, act as “Trojan Horse” to allow these intracellular bacteria to establish secondary infection foci, resulting in recurrent systemic infections (Tan et al., 2013). However, these same phagocytosis pathways could also be co‐utilised to allow antibiotics to be delivered to macrophages. Theoretically, nano‐carriers containing antibiotics are able to passively accumulate in infection foci via recognition and uptake by phagocytes. Upon phagocytosis of particulate drugs, the antibiotic payload has the potential to be delivered into the infected cells, therefore enhancing penetration and release of antibiotics inside the infected cells (Briones et al., 2008; Bakker‐Woudenberg et al., 2004; Pinto‐Alphandary et al., 2000; Schiffelers et al., 2001).

Furthermore, biofilm‐associated antimicrobial resistance associated with ESKAPE pathogens like *P*. *aeruginosa* and K. pneumoniae is also resistant to antibiotics (Rasamiravaka et al., 2014; Vuotto et al., 2017). Nano‐carriers could act as a protective coat, shielding against interactions and minimizing inactivation of drug by biofilm compartments and resident enzymes. In this scenario, nano‐based drug delivery systems (DDSs) are a promising way to treat either intracellular and biofilm‐forming ESKAPE pathogen infections. Diverse nano‐formulations, such as liposomes and polymeric nanoparticles (NPs), have been developed and employed for the delivery of antibiotics to difficult‐to‐treat bacteria (Table 1).

**Table 1:** Nano‐based drug delivery systems for ESKAPE pathogens.

Nanoparticles platform	Loaded antibiotics	Target pathogen(s)	Outcomes	Reference
Pyochelin‐based PEGylated liposomes	Cefepime, imipenem, and ceftazidime	P. aeruginosa (MDRPa)	Killed MDRPa within infected HaCaT keratinocytes without any cytotoxic effects at four times MIC concentrations after 72 h	Pushparaj Selvadoss et al. (2017)
DPPC, cholesterol liposomes	Ciprofloxacin	*P*. *aeruginosa*	The ciprofloxacin concentration required to achieve similar biofilm inhibition was 125‐fold lower compared with free ciprofloxacin	Bandara et al. (2016)
DA liposomes	Chloramphenicol	S. aureus (MRSA)	An inhibition zone about twofold higher, compared with free drug, was achieved by DA liposomes. DA liposomes augmented antibacterial activity on keratinocyte‐infected MRSA	Hsu et al. (2017)
Liposome, made by phosphatidylcholine, cholesterol, Tween 80, and stearylamine	Amikacin	K. pneumoniae	The liposome was able to deliver entrapped phage inside macrophages, caused 94.6% killing of intracellular *K*. *pneumoniae* and showed synergistic activity to eradicate mature biofilm of *K*. *pneumoniae*	Singla et al. (2016)
PLGA nanoparticles	Amikacin	*P*. *aeruginosa*	Particles penetrated through the entire biofilm thickness, more effective than free drug in biofilm eradication	Sabaeifard et al. (2017)
Mesoporous silica nanoparticles	Gentamicin	*S*. *aureus*	The inflammation‐related gene expression in infected preosteoblast or macrophage was downregulated significantly after treatment by the antibiotic loaded nanoparticles	Yang et al. (2018)
PpZEV‐NPs, made by PEG‐PLGA, Eudragit E100, and chitosan derivative	Vancomycin	*S*. *aureus* (MRSA)	PpZEV‐NPs showed better antimicrobial activity than free vancomycin against intracellular MRSA	Pei et al. (2017)
CSNPs	Cefazolin	*K*. *pneumoniae* *P*. *aeruginosa*	Excellent antimicrobial potential of cefazolin‐loaded CSNPs was demonstrated against multidrug‐resistant *K*. *pneumoniae* and *P*. *aeruginosa*	Jamil et al. (2015)

CSNPs, chitosan nanoparticles; DA, deoxycholic acid; DPPC, dipalmitoylphosphatidylcholine; *K*. *pneumoniae*, *Klebsiella pneumoniae*; MDRPa, multidrug‐resistant *P*. *aeruginosa*; MIC, minimum inhibitory concentration; MRSA, methicillin‐resistant *S*. *aureus*; PLGA, poly(lactide‐co‐glycolide); *P*. *aeruginosa*, *Pseudomonas aeruginosa*; *S*. *aureus*, *Staphylococcus aureus*.

#### Liposomes

Liposomes, first explored in the 1960s, represent the most developed DDS platform (Deamer, 2010; Düzgüneş et al., 2005). They are spherical vesicles composed of phospholipid bilayers surrounding an aqueous core. Within the structure, liposomes are capable of carrying hydrophilic drugs like aminoglycosides in their core or embed hydrophobic drugs like beta‐lactams into the bilayers (Drulis‐Kawa et al., 2010; Jones, 1995). Various studies have suggested that liposomal formulation could significantly enhance the antimicrobial effect of antibiotics against intracellular ESKAPE pathogens. For example, Pumerantz et al. (2011) demonstrated that vancomycin‐loaded liposomes composed of cholesterol and 1,2‐distearoyl‐sn‐glycero‐3‐phosphocholine a significant intracellular reduction in MRSA colony forming units compared with free drug. Additionally, liposome formulations have also been shown to exhibit enhanced therapeutic effects towards biofilm‐forming P. aeruginosa. As reported by Alhajlan et al. (2013), the biofilm‐forming strains of P. aeruginosa were more susceptible to clarithromycin‐encapsulated liposome than the free drug. However, some drawbacks were revealed in the development of antibiotic‐loaded liposomes, such as instability of the vesicles and low drug encapsulation. Therefore, polymeric NPs have been developed as alternative nano‐formulation platforms to improve stability and drug loading.

#### Polymeric nanoparticles

Polymeric NPs are prepared from natural or synthetic polymers with a size between 10 and 1000 nm. They can be a polymeric matrix with homogenous drug distribution or nano‐capsules with drug entrapped in a polymeric shell (Kreuter, 1991; Soppimath et al., 2001). Because of the development of advanced nanotechnologies and new polymers, NPs are currently the subject of extensive research as passive macrophage targeting agents, showing an ideal way to target macrophages to deliver therapeutic effects (Bains et al., 2016; Spence et al., 2015). Chitosan, a representative of commonly investigated natural polymers, has been widely used for mucoadhesive drug delivery and gene transfection (Kim et al., 2014). Our group previously reported that tobramycin‐loaded NPs, made of chitosan, and then functionalized with dornase alfa, demonstrated enhanced antibacterial effects on P. aeruginosa, DNA degradation, and improved NP penetration of CF sputum (Deacon et al., 2015). *O*‐carboxymethyl chitosan NPs loaded with tetracycline were reported by Maya et al. (2012), where the NPs were sixfold more effective in killing intracellular S. aureus compared with tetracycline alone in HEK‐293 and differentiated THP‐1 macrophage cells proving it to be an efficient nanomedicine to treat intracellular S. aureus infections. Another report illustrated that gentamicin‐loaded chitosan/fucoidan NPs were developed to provide multiple antimicrobial capabilities against K. pneumoniae, representing an improvement in antimicrobial efficacy (Huang et al., 2016). Synthetic polymers have been used for targeted delivery of antibiotics to macrophages for the treatment of intracellular ESKAPE infections. Among them, poly(lactide‐co‐glycolide) (PLGA) is a nontoxic, biodegradable, and biocompatible copolymer (Rajeev, 2000). Our previous work demonstrated that gentamicin‐loaded PLGA NPs were able to significantly eradicate biofilm‐forming P. aeruginosa, improving the antimicrobial effects of gentamicin (Abdelghany et al., 2012). Recently, our group also reported that using an emulsification‐solvent evaporation method, PLGA NPs containing gentamicin could effectively eliminate intracellular K. pneumoniae in a bacteria and macrophage coculture model (Jiang et al., 2018). Another study has shown that the effective delivery of encapsulated antibiotics (gentamicin and nafcillin) into cells augmented their therapeutic activity against intraphagosomal S. aureus (Imbuluzqueta et al., 2011, 2012; Pillai et al., 2008). Furthermore, nano‐formulated DDSs are also used for other intracellular bacteria, such as *M*. *tuberculosis* (Dollnellan et al., 2017). For example, PLGA NPs have been previously explored to deliver isoniazid into *M*. *tuberculosis* infected murine bone marrow‐derived macrophages (Faria et al., 2012).

### Nano‐formulation of antimicrobial treatments

The emergence of MDR ESKAPE pathogens has driven the urgent need for novel alternatives to antibiotics and has made researchers consider nano materials themselves as antibacterial agents. Silver has been known for its antibacterial activity since the ancient Egyptians and Greek periods, but it was only recently discovered that silver ion (Ag^+^) has broad‐spectrum antimicrobial activity (Klassen, 2000). With the development of nanotechnology, it is now possible to use silver NPs against infections caused by ESKAPE pathogens. For instance, Bankalgi et al. (2016) demonstrated that phenolics‐coated silver NPs showed strong antibacterial effects against Gram‐negative *P*. *aeruginosa* and Enterobacter aerogenes. Furthermore, Kedziora et al. (2016) demonstrated that silver nanoform complexed with amorphous TiO_2_ exhibits antimicrobial efficacy against *S*. *aureus* and K. pneumoniae. Although silver NPs show potential in antimicrobial applications, some adverse events associated with the use of these NPs, such as reactive oxygen species production, could damage the host cell. As an alternative, gold (AuNPs), which possess low cytotoxicity, have also been investigated as antimicrobial agents. It was reported that AuNPs functionalized with ampicillin elicited effective broad‐spectrum bactericidal activity against the ESKAPE pathogens, *P*. *aeruginosa* and E. aerogenes (Brown et al., 2012). Additionally, Zhao et al. (2013) reported that pyrimidinethiol‐modified AuNPs demonstrated synergistic antimicrobial effects against MDR *E*. *faecium*, MDR P. aeruginosa, MRSA, MDR *K*. *pneumoniae*, and MDR *A*. *baumannii*. Thus, the application of AuNPs seems to hold much potential for the treatment of MDR ESKAPE infectious diseases, but further developments such as manufacturability and pharmacokinetics must be addressed.

Some studies demonstrated other NP formulations that were capable of eliminating ESKAPE pathogens. For example, Friedman et al. (2011) have described a nitric oxide‐releasing NP with efficacy against not only MRSA and *A*. *baumannii* but also all the clinical isolates of *Streptococcus pyogenes*, *Enterococcus faecalis*, *K*. *pneumoniae*, and P. aeruginosa. Similarly, another report demonstrated that nitric oxide‐releasing silica NPs could be utilized as novel antibacterial agents against intracellular P. aeruginosa in L929 mouse fibroblasts (Hetrick et al., 2008). Wu et al. (2013) reported magnetic reduced graphene oxide NPs functionalized with glutaraldehyde, which provided rapid and effective killing of up to 99% of S. aureus. Additionally, Jones et al. (2008) demonstrated that ZnO NPs have a wide range of antibacterial effects against a number of microorganisms, having significantly higher antibacterial effects on S. aureus than other metal NPs.

### Antibody drug conjugation treatments

**Figure 1:**
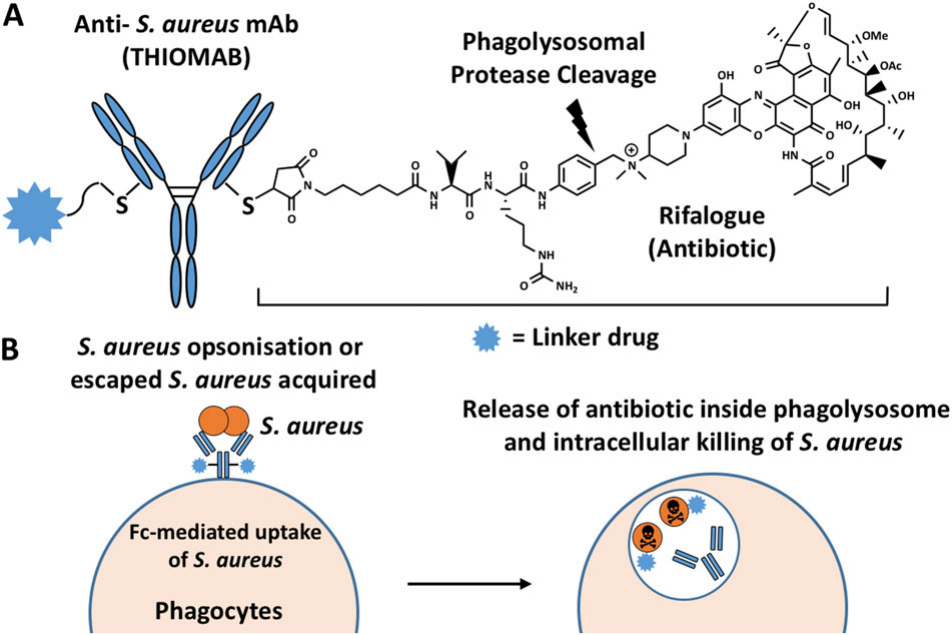
A. Model of antibody antibiotic conjugate (AAC). The AAC consists of an anti‐*S*. *aureus* antibody covalently linked via the introduced cysteines to an antibiotic using a cathepsin‐cleavable linker containing a novel quaternary ammonium salt. B. Mechanism of AAC action. When AAC opsonized bacteria are taken up by host cells, intracellular proteases cleave the linker and readily release the antibiotic in its active form to kill *S*. *aureus*.

Antibody drug conjugates developed as “magic bullets” have been successfully developed for cancer treatment, consisting of monoclonal antibodies specifically targeted to antigen‐expressing tumour cells with cytotoxic drug payloads (Casi et al., 2012; Wang et al., 2015). This approach has now been exploited for treatment of infectious diseases – antibody antibiotic conjugates (AACs). In AACs, an antibiotic is conjugated to an antibody to target the bacteria of interest. Recently, scientists at Genentech have developed an AAC called THIOMAB™ antibiotic conjugate aimed at the treatment of intracellular MRSA. This AAC consists of an antibody that binds the surface of S. aureus (wall‐teichoic acids and pathogen‐specific polyanionic glycopolymers) and carries the potent rifalogue antibiotic (Lehar et al., 2015). Once the bacterium/AAC complex is internalized by host cells, the host‐resident proteases release the antibiotic payload, so that it can act directly at the site of intracellular infection (Fig. 1). These researchers have demonstrated that this AAC could significantly eradicate intracellular S. aureus infections with a superior therapeutic effect than vancomycin. This therapeutic (DSTA‐46375) is currently in phase I clinical trials. AACs have the potential to modify a broad‐spectrum antibiotic into a pathogen‐specific antibiotic as a result of the antibody used, therefore minimizing side effects such as ototoxicity and nephrotoxicity. However, AACs are complex molecules, so further developments in design and manufacturing are required for application against difficult‐to‐treat ESKAPE bacterial infections.

## Perspective

Given the increasing prevalence of antibiotic resistance, the treatment of ESKAPE pathogens is becoming increasingly more challenging. Intracellular ESKAPE pathogens represent a group of bacteria that are particularly difficult to treat as a result of their intracellular residual location. Pathogen‐harbouring phagocytes effectively shield the intracellular bacteria from antibiotics, resulting in difficulties in eradicating the infection as well as limitations in clinical treatment options. Antibiotic‐loaded DDS may represent an exciting approach for the treatment for intracellular bacteria in the future using existing and novel antibiotics. With the developments of DDS, intracellular accumulation of these poorly cell‐permeable drugs has been circumvented, leading to an enhancement of antimicrobial activities.

A key concept in the strategy of employing nano‐technological delivery systems for ESKAPE intracellular infections is that the pathogens and the NPs tend to accumulate in the same cell – the professional phagocytes of the reticuloendothelial system. It may be possible in the future to explore more “active” targeting strategies, exemplified by the current interest in AACs.

In conclusion, through reformulation of existing antibiotics, it may be possible to extend the useful life span of these drugs and their ability to treat dangerous intracellular infections.

## Funding Information

This work was funded in part through Biotechnology and Biological Sciences Research Council Award BB/P006078/1 and Engineering and Physical Sciences Research Council Award EP/M027473/1 and a Royal Society Industrial Fellowship to C. J. Scott.

## Conflict of Interest

None declared.
